# Supplementary material for: Strategies to Cope with Inferior Long-Term Photostability of Bentonite Polyolefin Nanocomposites
Source: Polymers (Basel). 2024 Feb 17;16(4):535. doi: 10.3390/polym16040535 (PMC10893414; doi:10.3390/polym16040535)
Supplement: Supplementary file 1 [file polymers-16-00535-s001.zip › polymers-2862983-supplementary.pdf]

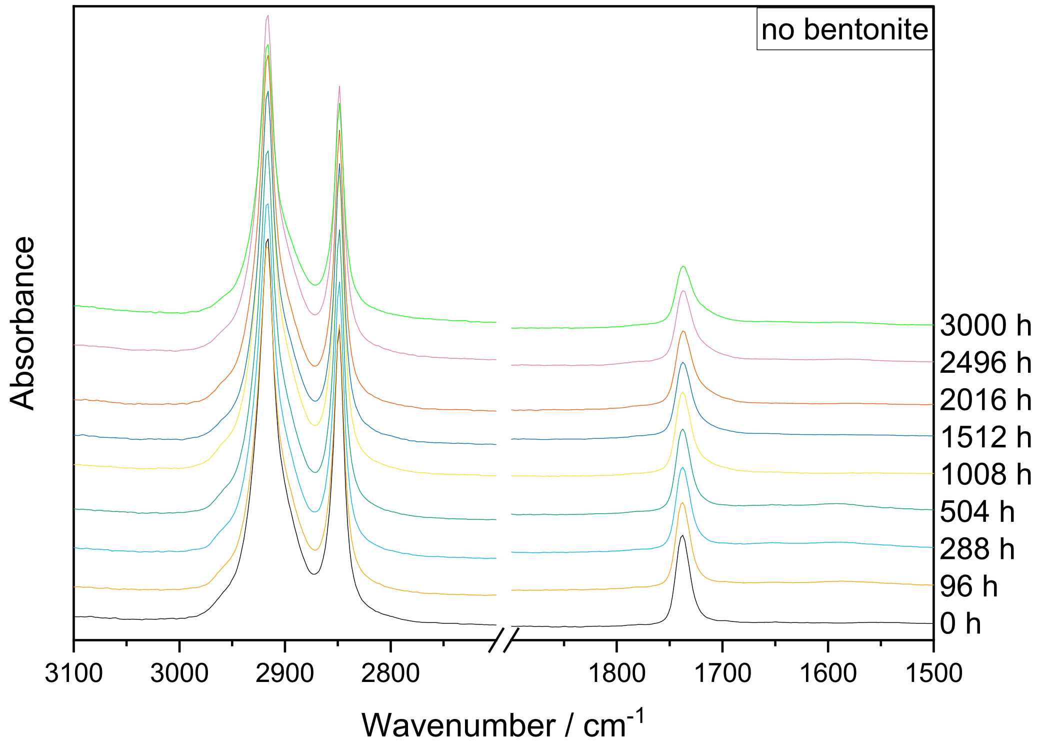

Figure S1. FTIR-spectra of bentonite-free composite during weathering.

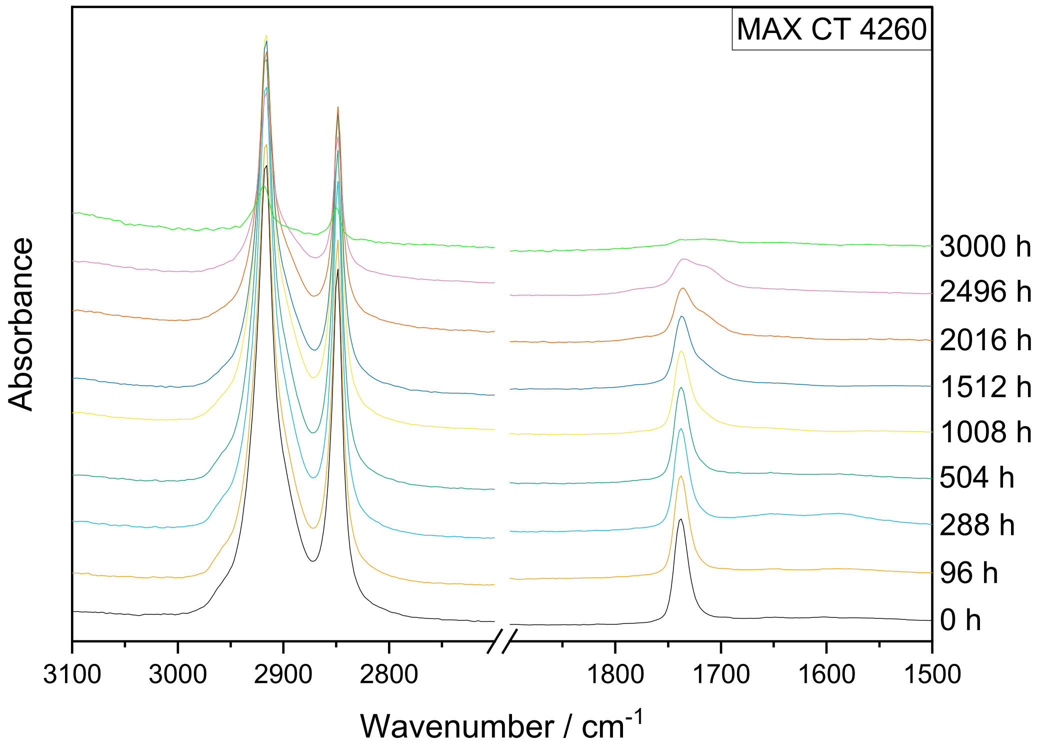

Figure S2. FTIR-spectra of MAX CT 4260 composite during weathering.

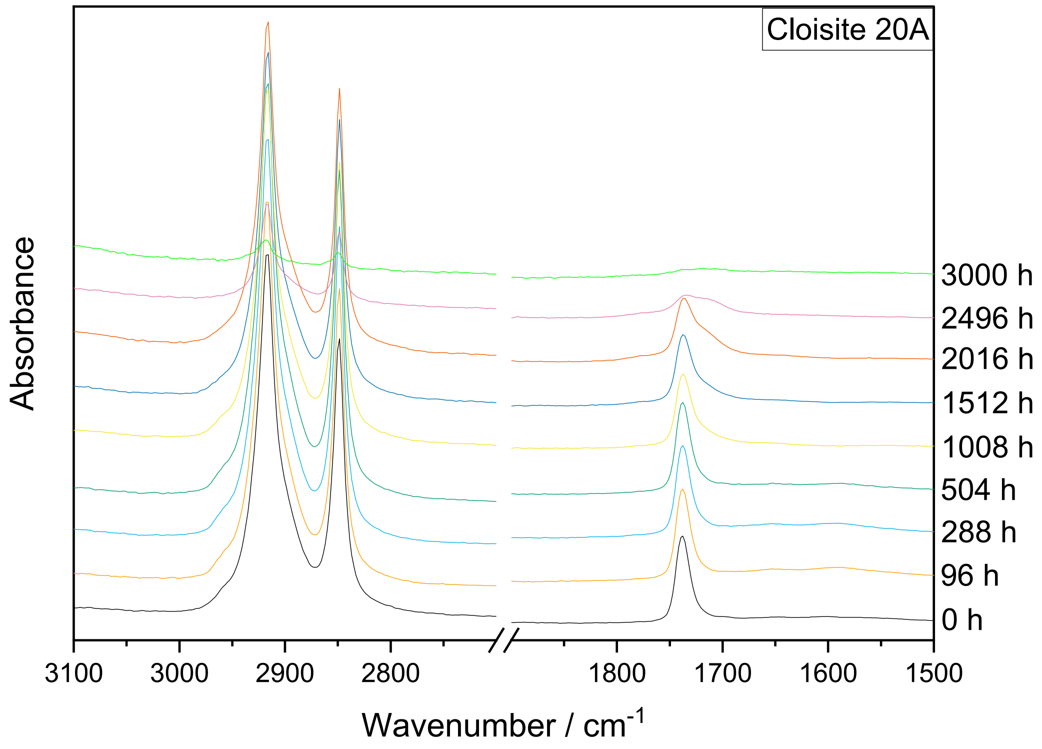

Figure S3. FTIR-spectra of Cloisite 20A composite during weathering.

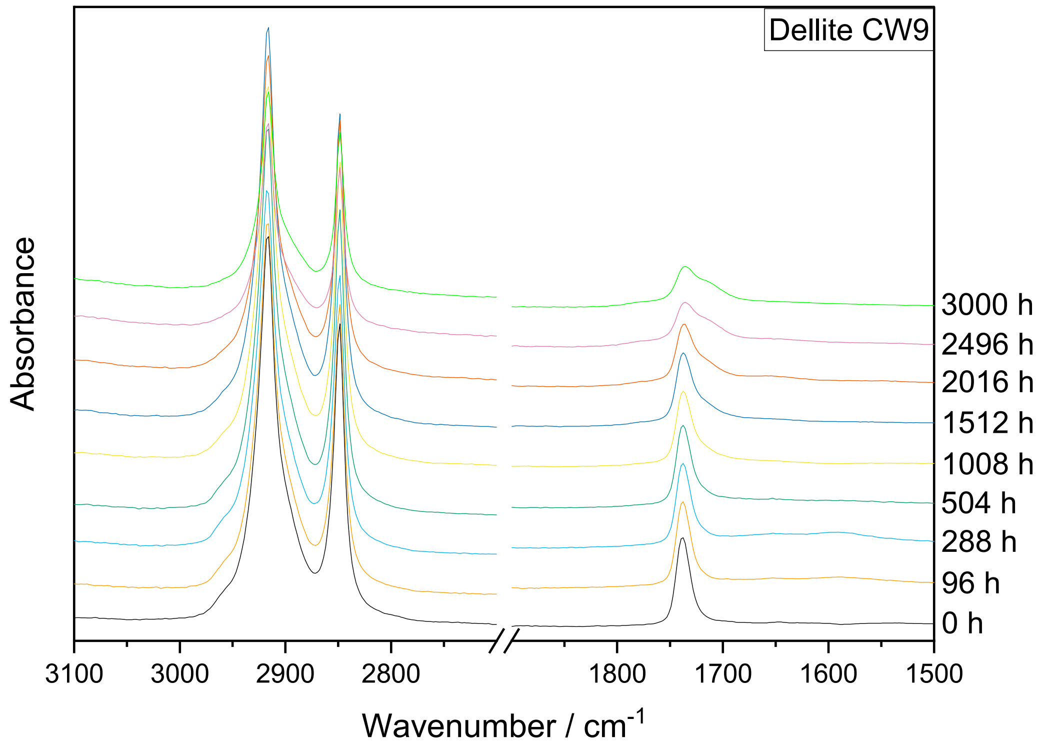

Figure S4. FTIR-spectra of Dellite CW9 composite during weathering.

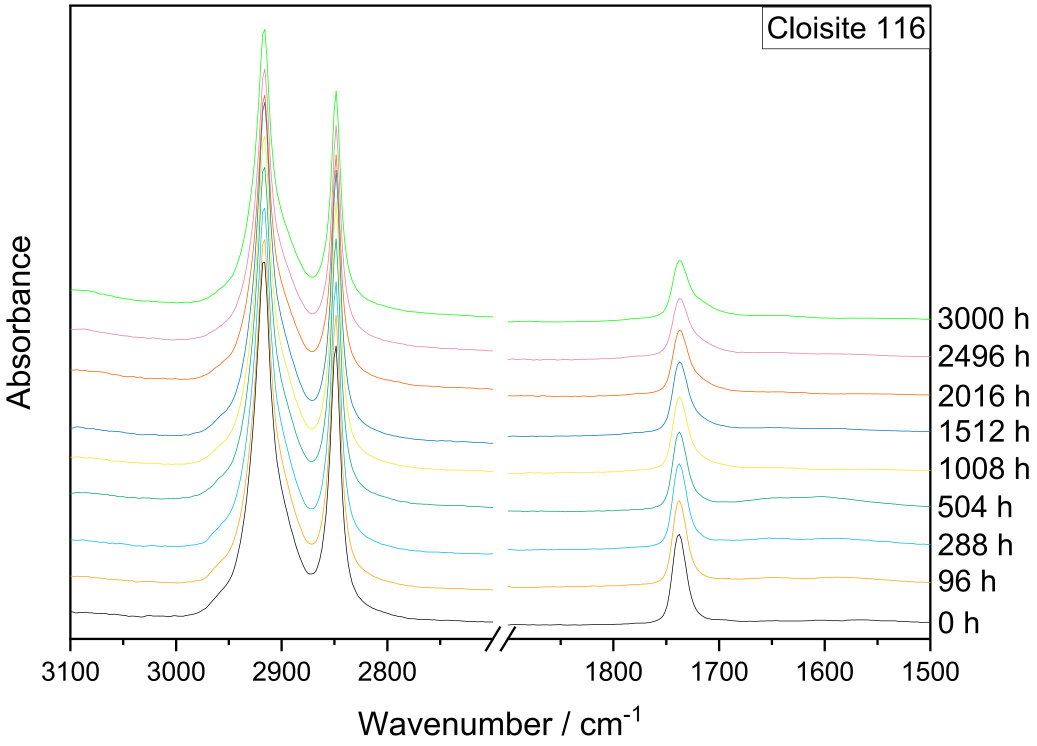

Figure S5. FTIR-spectra of Cloisite 116 composite during weathering.

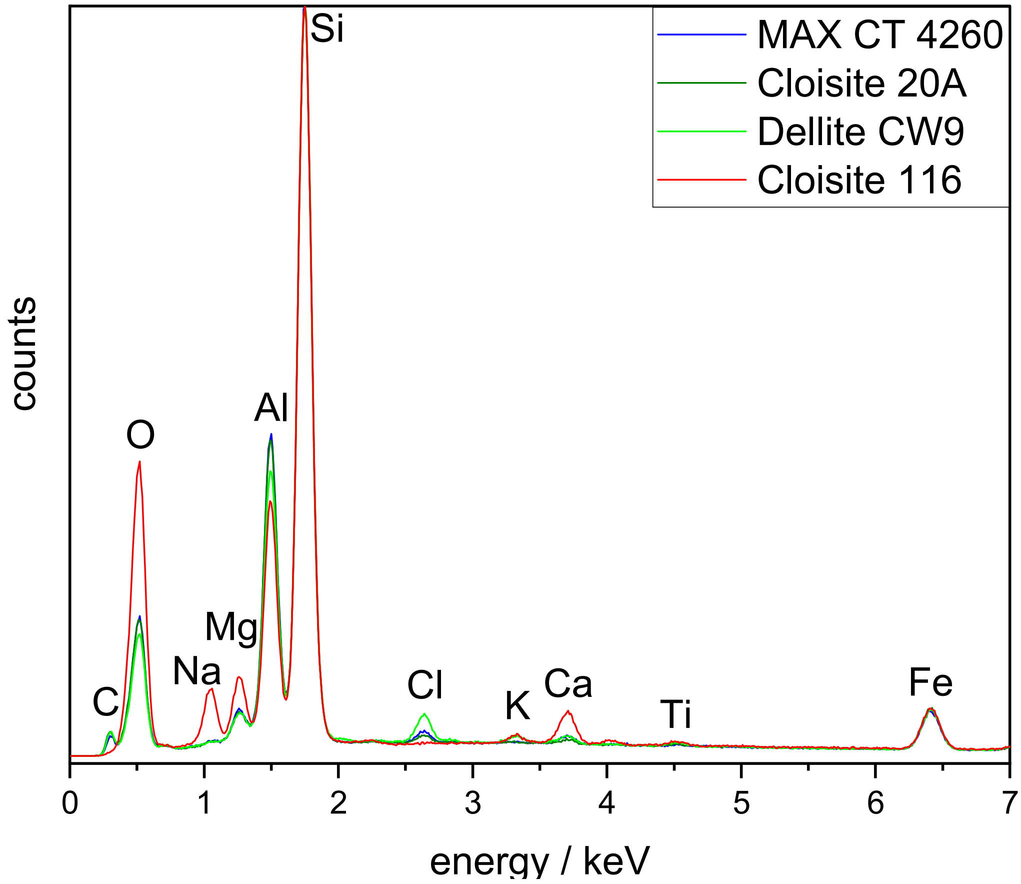

Figure S6. SEM-EDX spectra of bentonite products.
